# A Taxonomy and Archetypes of AI-Based Health Care Services: Qualitative Study

**DOI:** 10.2196/53986

**Published:** 2024-11-27

**Authors:** Marlene Blaß, Henner Gimpel, Philip Karnebogen

**Affiliations:** 1 FIM Research Center for Information Management University of Hohenheim Branch Business & Information Systems Engineering of the Fraunhofer FIT Stuttgart Germany; 2 FIM Research Center for Information Management Branch Business & Information Systems Engineering of the Fraunhofer FIT University of Applied Sciences Augsburg Augsburg Germany

**Keywords:** healthcare, artificial intelligence, AI, taxonomy, services, cluster analysis, archetypes

## Abstract

**Background:**

To cope with the enormous burdens placed on health care systems around the world, from the strains and stresses caused by longer life expectancy to the large-scale emergency relief actions required by pandemics like COVID-19, many health care companies have been using artificial intelligence (AI) to adapt their services. Nevertheless, conceptual insights into how AI has been transforming the health care sector are still few and far between. This study aims to provide an overarching structure with which to classify the various real-world phenomena. A clear and comprehensive taxonomy will provide consensus on AI-based health care service offerings and sharpen the view of their adoption in the health care sector.

**Objective:**

The goal of this study is to identify the design characteristics of AI-based health care services.

**Methods:**

We propose a multilayered taxonomy created in accordance with an established method of taxonomy development. In doing so, we applied 268 AI-based health care services, conducted a structured literature review, and then evaluated the resulting taxonomy. Finally, we performed a cluster analysis to identify the archetypes of AI-based health care services.

**Results:**

We identified 4 critical perspectives: agents, data, AI, and health impact. Furthermore, a cluster analysis yielded 13 archetypes that demonstrate our taxonomy’s applicability.

**Conclusions:**

This contribution to conceptual knowledge of AI-based health care services enables researchers as well as practitioners to analyze such services and improve their theory-led design.

## Introduction

We are entering a new health era—an era of digital health defined by innovative medical solutions and an explosion of health-related data [1]. Fueled by these amounts of data, forecasts indicate that artificial intelligence (AI) will soon exceed today’s capabilities in supporting and transforming health care systems [2-4]. There is reason to believe that AI will improve health care at various stages, ranging from disease detection to treatment [5], so much so that it promises to facilitate a new generation of health care services [6]. The proliferation of AI promises to be a panacea for our era’s multiple health care issues, including the ongoing fight against the COVID-19 pandemic (eg, studies by Chung et al [7] and Krämer et al [8]), the long-term health complaints of an aging population (eg, study by Velazquez et al [9]), and the ever-increasing rates of chronic diseases (eg, study by Sekandi et al [10]). It is unlikely that AI will live up to its hype of being a miracle cure for all health care–related problems, as its implementation is not without hurdles. Especially, privacy concerns due to the sensitive nature of health-related data and ethical considerations like the risk of biased algorithms leading to unequal health care outcomes pose significant challenges and require careful navigation.

Nevertheless, it has the significant potential of not only disrupting old and dysfunctional services but also providing new means of managing and perhaps resolving various health care challenges [2,11,12]. Already, the increasing reliance on AI is changing the nature of health care services. Indeed, AI is joining the established actors in health care services [13] and transforming the ways in which physical, mental, or social well-being is maintained or restored [14]. When we speak of AI in this context, it serves as an umbrella term for a broad set of algorithms [15-17] that use data to perform cognitive functions previously limited to humans [18,19].

Health care companies have included AI in their service provisions, which has led to ever-increasing investments in the development of AI-based health care services [20]. Likewise, the number of published research papers debating the role of AI in health care has gone up significantly, and it has done so in a broad range of disciplines. Despite this multilateral fascination with AI-based health care services, however, there is a notable and rather lamentable lack of conceptual insights into the design characteristics of AI-based health care services, which is why there is still no clear understanding of its general properties and potential.

To date, research has mainly focused on investigating the potential of individual AI applications in the health care sector [12,21-23]. Yet to be derived from these, however, is an overarching structure with which to classify the various real-world phenomena. In the absence of a clear and comprehensive taxonomy, there has been no consensus on AI-based health care service offerings, what they have in common, and how they differ. This lack of a shared understanding of AI services limits any further analysis of how AI may affect the health care sector, which in turn limits the ability of researchers to stay abreast of the life-changing and, indeed, life-saving developments in this domain [5,17]. Thus, this study sets out to answer the following research question: What are the design characteristics of AI-based health care services?

To answer this, we propose a multilayered taxonomy created in accordance with both the well-established method of taxonomy development by Nickerson et al [24] and the recent extension by Kundisch et al [25]. By exploring both empirical-to-conceptual (E2C) and conceptual-to-empirical (C2E) iterations, we pursued a bilateral development for the structure of our taxonomy, deductively from real-world examples and inductively from a structured literature review. Through 4 iterations, we identified 10 key dimensions and their respective characteristics, all of which we structured into 4 perspectives: agents, data, AI, and health impact. We also performed a thorough review of the current literature, particularly the 28 most relevant studies, and considered their insights with regard to 268 AI-based health care services. We then conducted a careful evaluation of our taxonomy, as proposed by Kundisch et al [25].

The taxonomy and the archetypes of AI-based health care services are of theoretical as well as practical value. With regard to the former, this study can provide a helpful frame of reference for the emerging field of digital transformation in health care by outlining and structuring the general features of AI-based health care services. With regard to the latter, our taxonomy makes it possible to map the field of AI-based health care services, assess their possibilities in a competitive field, and offer guidance to health care entrepreneurs.

## Methods

### Overview

To identify the defining features of AI-based health care services as well as their corresponding archetypes, we formulated a bilateral methodological approach to develop our taxonomy in accordance with both the guidelines of Nickerson et al [24] and their extension by Kundisch et al [25]. In doing so, we achieved our dual aim of creating a versatile set of tools for the analysis of both conceptual and empirical observations. Taxonomies comprise various layers, which in turn include multiple dimensions, which can again contain diverse characteristics [24]. They are frequently used (eg, Fischer et al [26] and Gimpel et al [27] to show how different concepts are connected or to investigate their relationships [28].

### Defining Preliminary Conditions

As proposed by Kundisch et al [25], we began our taxonomy development by specifying the central phenomenon under investigation, that is, AI-based health care services. To pay due attention to all of their various design characteristics as well as those of our 2 target groups—researchers examining the field of AI-based health care services and practitioners in the health care sector who are already integrating or aim to integrate AI in their service offerings—we define the meta-characteristic [24], that is, the design characteristics of AI-based health care services.

In line with the best practices of Nickerson et al [24], we then define our objective and subjective ending conditions, both of which are checked after each iteration to assess whether the taxonomy development process is completed [24]. Nickerson et al [24] identified a set of ending conditions that can be applied or adapted to the taxonomy development process, four of which we selected for this study: (1) all objects of our dataset have been examined, (2) no new dimension or characteristics were added in the last iteration, (3) every dimension is unique and not repeated, and (4) at least one object from our dataset is classified as having every characteristic of every dimension [24]. Furthermore, we concur with Nickerson et al [24] regarding the subjective ending conditions in that the taxonomy should be concise, robust, comprehensive, extendible, and explanatory [24].

### Taxonomy Development Process

Our taxonomy development process consists of 4 iterations. Nickerson et al [24] propose 2 development approaches to this development. First, inductive, which is to say E2C, and second, deductive, which is to say C2E [24]. These 2 approaches are to be executed consecutively in several iterations. Table 1 depicts details on this taxonomy development process, including the development approach chosen for each iteration, the objects in question, the significant changes, and the evaluation of the ending conditions.

**Table 1:** Details of the iterative taxonomy development process.

Iteration	App^a^	Objects	Major changes	Ending conditions
1	E2C^b^	25 AI^c^-based health care services randomly chosen from our sample	Identification of 3 perspectives with 10 dimensions and 35 characteristics in total	Objective and subjective ending conditions not met: not all objects were examined not robust because it is not grounded in theory, and not concise because of the many dimensions
2	C2E^d^	19 papers from a structured literature review	Addition of 1 perspective with 2 new dimensions and 5 new characteristics; reordering of the dimensions to match the perspectives	Objective and subjective ending conditions not met: not all objects were examined not all dimensions are unique not concise due to the many dimensions
3	E2C	25 AI-based health care services randomly chosen from our sample	Abandonment of 1 dimension; replacement of 2 dimensions due to significant overlap; modification of multiple characteristics	Objective ending conditions not met: not all objects were examined
4	E2C	The remaining sample of 218 AI-based health care services	No further modification	All ending conditions were met: objective ending conditions (1)-(4) are met the authors have achieved consensus and agree that the subjective ending conditions are met

^a^App: development approach.

^b^E2C: empirical-to-conceptual.

^c^AI: artificial intelligence.

^d^C2E: conceptual-to-empirical.

In our first iteration, we chose an E2C approach since this is generally agreed to be the best practice when a significant amount of data can be used to examine a specific research field. To collect the necessary empirical data when making our selection of AI-based health care services, we followed a similar approach to that of Labes et al [29] and Fischer et al [26]. In doing so, we were aware of the challenge of keeping up with the fast-changing field of AI in health care, which is why we identified so-called “top lists” that consolidate the most promising AI companies in health care. After compiling an initial list with the help of several internet search engines, using terms such as “top AI health care companies,” “ranking AI companies in health care,” and “top list health care and AI,” we synthesized our results into a single integrated list of AI-based health care services derived from 6 data sources. Our sample only included services that describe an activity involving at least 2 entities with different roles, for example, a patient and a health care professional, where both use their resources, be it information or technology [30], in a collaborative process that serves their mutual benefit. Furthermore, it was not our aim to compile a complete list of all existing health care start-ups that use AI in their portfolio. Instead, we wanted to collect a sample that was as representative and generalizable as possible to serve as a basis for the taxonomy development. For this reason, we used 6 different top lists as a data source, which are based on various evaluation criteria such as growth, funding, and global impact. Furthermore, we use top lists from the years 2020 and 2023 to also cover a temporal variance. This ensures that our sample reflects sufficient variance and can be considered representative. The final list comprised 268 AI-based health care services. Distinct subsamples were used in iterations 1, 3, and 4. Tables 2 and 3 illustrate the data sources as well as exemplary services.

**Table 2:** Empirical-to-conceptual data used for our taxonomy development.

Considered top lists	Exemplary firms (services)
Forbes [31]	Bioformis (Biovitals); Karius (Karius-Bloodtest); Viz.ai (Stroke Monitoring)
Medical startups [32]	Babylon Health (Ask Babylon, Healthcheck); Suki (Digital Assistant; Speech Service)
Startus [33]	Roam Analytics (Roam Health Knowledge Graph); Sopris Health (Sopris Assistant); Artelus (DRISTi, Hansanet)
CBInsights [34]	Mindstrong (Mindstrong Service); Freenome (Cancer detection); PathAI (Imaging Service)
QWayHealth [35]	Arterys (Cardio AI, Lung AI) Caption Health (Caption AI); Corti (Audia)
Medical futurist [36]	Google Deepmind (Eye Disease Scan); Oncora Medical (Patient Care, Analytics); CloudMedX (AskSophie; Decision Point)
Total services, n	268

**Table 3:** Conceptual-to-empirical data used for our taxonomy development.

Activity	Details
Search string	(“artificial intelligence” OR “AI” OR “machine learning” OR “deep learning”) AND (“healthcare” OR “health care” OR “clinical” OR “medicine”) AND (“service” OR “application”)
Databases sources	Web of Science; forward and backward search
Results	20 papers found in the initial search; 8 papers found in the forward and backward searches [37]
Papers found	Agrawal et al [15]; Ågerfalk [17]; Reis et al [23]; Fischer et al [26]; Gimpel et al [27]; Bao et al [37]; Baars and Kemper [38]; Bardhan et al [39]; Brynjolfsson and Mitchell [40]; Chang [41]; Hansen and Baroody [42]; Hofmann et al [43]; Hunke et al [44]; Iansiti and Lakhani [45]; Mantzana et al [46]; Reddy et al [47]; Schuetz and Venkatesh [48]; Tan et al [49]; Thompson et al [50]; Wani et al [51]; Weglarz [52]; Antoniadi et al [53]; Shamshirband et al [54]; Ali et al [55]; Qadri et al [56]; Karatas et al [57]; Shatte et al [58]; Bindra and Jain [59]
Total papers, n	28

In the first iteration, we analyzed a random sample of 25 AI-based health care services to derive relevant dimensions and characteristics. In the second iteration, we followed a C2E approach informed by the insights of a structured literature review [60]; Tables 2 and 3 contain the details. With this 2D approach to data collection from practical and academic data sources, we were able to account for the multiple developments in the rapidly expanding field of AI-based health care services. To broaden our understanding accordingly and heed the lessons learned from the research already conducted, we performed a literature review on the Web of Science. We used an iterative search process for the literature review. Initially, we had a very narrow search string to gain an initial understanding of the topic, which we later expanded again to ensure a more comprehensive literature review. For the final search, we limited our search to papers published between 2015 and 2023 in the English language and excluded book chapters or proceeding papers. By using the exact search string “(“artificial intelligence” OR “AI” OR “machine learning” OR “deep learning”) AND (“healthcare” OR “health care” OR “clinical” OR “medicine”) AND (“service” OR “application”),” we were able to exclude any literature irrelevant to our study. This left us with 9318 relevant results. Furthermore, 2 members of the research team (MB and PK) then analyzed the title and abstract of each paper. After that, all those who did not directly address the topic under investigation were excluded. Since reliability was a guiding principle of this process, the team screened and classified a random sample of 30 papers in close collaboration. After that, 30 papers were screened independently, and their classifications were compared to certify the analytical adequacy. All subsequent papers were screened individually by one of the team members. Speaking in numbers, this meant that from the initial set of 9318 papers, 276 had their full text analyzed, and 20 of these were considered for our taxonomy. In addition, 8 papers were included through forward and backward searches, which brought the total to 28 papers.

For the third and fourth iterations, we again followed the E2C approach with 25 randomly selected AI-based health care services and 218 remaining from our initial sample. After the fourth iteration, the team agreed that the objective and subjective ending conditions were all met.

### Demonstration and Evaluation Process

As Kundisch et al [25] pointed out, a rigorous demonstration and evaluation of a newly developed taxonomy require a 2-step approach. In the first step, 13 researchers with a domain-specific background in AI or health care, or indeed both, classified multiple real-world services by using our taxonomy. These demographics were selected because they constitute the intended future users of our taxonomy. We measured the agreement among the individual classifications by means of the percentage agreement and Fleiss κ [61]. The latter was chosen by virtue of the fact that it can be used to determine the degree of agreement between the assessments of more than 2 raters, which is to say that it works as an extension to Cohen κ [62], provided that the number of raters per assessed unit remains constant (described in detail in “Taxonomy Evaluation” in Multimedia Appendix 1). The unweighted κ [61] is determined, that is, each nonmatch is weighted equally. The calculation of κ [61] (
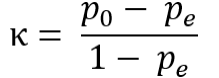
) results from the calculation of the relative agreement of the raters (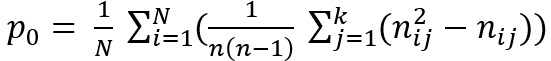) and the calculation of the probability of random agreement (
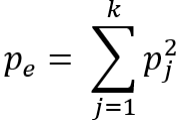
).

n describes the number of raters (n=13), N is the number of considered service offerings per rater, and k is the number of possible evaluation categories. Aside from being a well-established procedure to evaluate all manner of taxonomies, it allowed us to ensure that classifications made with our taxonomy are not too susceptible to personal bias [27,63].

In the second step, we classified all available services to learn more about the occurrence rate of each characteristic in our dataset. This was followed by cluster analysis, a statistical method of similarity analysis performed to identify groups of similar objects, so-called clusters, within a dataset [64-66]. The matching objects are grouped in such a way as to achieve high homogeneity within clusters and high heterogeneity between clusters [67]. In our case, this cluster analysis allowed us to identify archetypes of AI-based health care services by observing the three necessary specifications: (1) a proximity measure to calculate similarities or distances between objects, (2) a clustering method with underlying grouping methods or fusion algorithms, and (3) the order in which the clusters are split or merged. It is worth noting, however, that the choice of the clustering procedure is closely related to the question of the optimal number of clusters. To determine an appropriate number of clusters for AI-based health care services, we calculated several measures discussed in the literature (described in detail in “Clustering” in Multimedia Appendix 1). For the various numbers of clusters, each analysis is performed with an agglomerative hierarchical method that creates a corresponding cluster solution for any number of clusters between 1 and n, where n provides the number of services considered in the sample [68,69]. To this end, we chose the Ward algorithm [70] as our clustering algorithm and used the Manhattan distance as our distance measure, seeing as the latter can be applied to nominally scaled data and has proven to perform well in connection with the Ward algorithm [70,71]. To ensure the correct application of the distance measure, we first dichotomized and then standardized the underlying data by accounting for the number of characteristics per dimension, thus also ensuring that all dimensions are equally weighted.

### Ethical Considerations

This study did not require approval from an ethics review board, as it does not constitute human participants research. In accordance with the guidelines of the University of Hohenheim, ethical review is mandated only for research involving human participants, biological samples, or identifiable personal data. The focus of this study was on identifying the design characteristics of AI-based health care services, relying primarily on a review of existing literature and analysis of established AI-based health care services. Data from individuals were collected solely for the purpose of evaluating the proposed taxonomy, and as such, the study does not meet the criteria for human participants research as defined by the relevant policies of the University of Hohenheim (Satzung der Ethikkommission der Universität Hohenheim).

## Results

### A Taxonomy of AI-Based Health Care Services

#### Overview

Following the guidelines of Nickerson et al [24], we developed the taxonomy of AI-based health care services shown in Table 4. The taxonomy is predicated on the 4 perspectives of agents, data, AI, and health impact, and it comprises 10 essential dimensions structured in accordance with these 4 perspectives. However, whereas Nickerson et al [24] suggest that one should design all characteristics as mutually exclusive in order for a single characteristic to be observed per dimension at any one time [24], we instead decided to adhere to the design principles of common taxonomies [26,27,44]. Nonexclusive dimensions could be replaced with binary dimensions for each characteristic. However, this would drastically increase the size of the taxonomy and, thus, impair conciseness, which is why we decided to include nonexclusive dimensions. As a result, we are able to reflect on the complexity of AI-based services more appropriately while keeping the taxonomy parsimonious. Whether or not a dimension is mutually exclusive is also indicated in Table 4, where we introduce the dimensions and characteristics in detail.

**Table 4:** Taxonomy of AI^a^-based health care services.

Perspective and dimension		Characteristic	E^b^ or N^c^
**Agents**			
	Service recipient	Patient Health care professional	N
	Mode of interaction	AI-driven Recipient-driven	E
**Data**			
	Data generator	Patient Health care professional Object	N
	Data target	Patient Health care professional Environment	N
	Data type	Structured Unstructured	N
**AI**			
	Hardware agnostic	Yes No	E
	AI portfolio integration	Wrapped around product Wrapped around service Stand-alone solution	E
	AI capability	Recognizing Reasoning Predicting Decision-making Generating Acting	N
**Health impact**			
	Application area	Prevention Diagnostics Treatment and care Patient enablement	N
	Health benefit	Triage Physical Mental Social	N

^a^AI: artificial intelligence.

^b^E: mutually exclusive.

^c^N: mutually nonexclusive.

#### Agents Perspective

In this perspective, one looks at the entities involved in the provision of a service and the ways in which they interact during this service provision [46,71]. Therefore, this perspective has 2 dimensions, that is, the service recipient and the mode of interaction. The service recipient is the primary beneficiary of the service. Even though most health services ultimately aim to improve or maintain the health of patients, this category refers to the direct recipient of a given service [37]. Health care services can have various stakeholders [49], but during the development process of this study, we learned from our empirical iterations that only 2 potential entities profit directly from AI-based health care services, that is, the patient and the health care professional. How these entities are involved in the service communication and who triggers the information exchange is determined in the dimension mode of interaction. The interaction can be either AI-driven, meaning the service itself actively initiates interaction and requests information, or recipient-driven, meaning the exchange of information is triggered by the service user [27].

#### Data Perspective

In this perspective, one examines the data that are generated during a service. This perspective, then, is predicated on the foundation of the underlying AI algorithm. It comprises 3 dimensions: the data generator, the data target, and the data type. As Hunke et al [44] noted, analytics-based services are characterized by a data generator and a data target. A data generator describes the entity that actively generates the data, while the data target describes the source from which the data emerges [44]. Since this also applies to our topic of investigation, we incorporated these 2 dimensions into our taxonomy once we made the necessary adaptations to the new context of our study. For AI-based health care services, data can be generated either by the patient, the health care professional, or an object involved in the service (eg, a diagnostic device). These data contain information about the patient, the health care professional, or the patient’s environment, and it can take the shape of different data types, being either structured or unstructured [38,52]. Structured data are characterized by enforced composition to anatomic data types and can be directly processed, for example, into laboratory values in electronic health records [42]. In contrast, unstructured data have neither a structured format nor a conceptual definition, such as image or audio data.

#### AI Perspective

From this perspective, one can analyze how AI is integrated into health care services. It comprises 3 dimensions: hardware agnostic, AI portfolio integration, and applied AI capabilities. The dimension of hardware agnostic is based on our E2C iterations. It allows one to ascertain whether or not a service relies on specific hardware or specific platform resources, making the AI-based service either device-independent (“yes”) or device-dependent (“no”). AI portfolio integration indicates whether the AI is integrated into a new stand-alone solution, linked to an existing product, or connected to a current service that has already been provided [44]. The dimension of AI capabilities refers to the idiosyncratic characteristics of AI algorithms that enable cognitive tasks formerly performed by humans [15,17,18,40,43,45,48]. The identified tasks include recognizing patterns or concepts, reasoning about relationships among distinct variables, predicting future outcomes or conditions, deciding between discrete options, generating something new, or even just acting.

#### Health Impact Perspective

In this final perspective, one focuses on the delivery of an AI-based service and its impact on health care [47]. There are 2 dimensions to this: one must identify the exact application area in the health care sector as well as the health benefit achieved by the service. After all, the incorporation of AI in the provision of health care services can impact different application areas in the health care sector. By fostering healthy lifestyles or avoiding unhealthy habits, AI-based services can help humans prevent new diseases. They can also help medical professionals refine their disease diagnosis and improve their treatment and care of patients [50]. Furthermore, our empirical investigation of AI-based health care services has shown that AI techniques can empower patients or professionals to actively handle a disease or reduce the adverse impact of a disability. In addition, this positive impact is scalable to a broad range of health care domains, where AI-based health care services can leverage various health benefits. In accordance with the World Health Organization’s definition of health [14], AI-based health care services can impact the physical and psychological as well as the social aspects of health. Indeed, AI techniques can even refine medical triage to facilitate a more targeted treatment.

### Demonstration and Evaluation

To validate the robustness of classifications made with our taxonomy, we recruited 13 researchers in the fields of AI, health care, or both. The overall demonstration phase took 3 weeks and was analyzed by 2 of the authors (MB and PK). We tasked the 13 researchers to classify 4 service offerings from our dataset anonymously. In choosing the service offerings for the application of our taxonomy, we made sure to cover a wide range of characteristics to provide a holistic evaluation of the taxonomy. Each service provider supplied the participating researchers with a brief description of their service along with a few images to illustrate how it works. As a further quality control measure, we made sure that there could be no confusion about the task assigned to the participants by giving them descriptions of all the dimensions and characteristics that they would have to consider when using the taxonomy, and we tested the comprehensibility of the questions as well as the appropriateness of the supplementary texts in advance. As a result of these measures, the percentage agreement among all authors and concerning all dimensions was an excellent 93.4%. Complementarily, the overall percentage agreement among all participants was 88% [72]. Fleiss κ is 62%, indicating a “substantial” agreement among participants [73]. For a detailed overview of percentage agreement and Fleiss κ across all individual dimensions, see “Taxonomy Evaluation” in Multimedia Appendix 1 [61]. It is suffice it to say that we conclude the taxonomy to be concise, robust, comprehensive, extendible, and explanatory [24].

In the following phase, we classified all 268 health care services to reveal the absolute and relative frequencies of all characteristics. When analyzing the relative frequencies, as presented in Table 5, some significant observations can be made, such as the health care professional is a direct recipient in 70% (n=186) of services, and the service recipient triggers the exchange of information in 79% (n=211) of services. Also noteworthy is that 74% (n=198) of data generators are objects, such as wearable devices. In 95% (254) of services, the primary data target is the patient, while 70% (n=188) of the data underlying the service are unstructured. As for the role of AI, 84% (n=223) of services are hardware agnostic, which is to say that they do not require a specific device to deploy the service. Also of interest in this context is the statistic that 83% (n=222) of the services in our sample were offered as a stand-alone solution.

**Table 5:** Absolute and relative frequencies of the characteristics of 268 AI^a^-based health care services.

Perspective and dimension			Characteristic		Frequency (N=268), n^b^ (%^c^)
**Agents**					
	Service recipient	Patient Health care professional		96 (36) 186 (70)	
	Mode of interaction	AI-driven Recipient-driven		56 (21) 211 (79)	
**Data**					
	Data generator	Patient Health care professional Object		72 (27) 26 (10) 198 (74)	
	Data target	Patient Health care professional Environment		254 (95) 7 (3) 23 (9)	
	Data type	Structured Unstructured		95 (36) 188 (70)	
**AI**					
	Hardware agnostic	Yes No		223 (84) 44 (16)	
	AI portfolio integration	Wrapped around product Wrapped around service Stand-alone solution		22 (8) 23 (9) 222 (83)	
	AI capability	Recognizing Reasoning Predicting Decision-making Generating Acting		257 (96) 34 (13) 28 (10) 75 (28) 89 (33) 30 (11)	
**Health impact**					
	Application area	Prevention Diagnostics Treatment and care Patient enablement		57 (21) 163 (61) 83 (31) 16 (6)	
	Health benefit	Triage Physical Mental Social		25 (9) 231 (87) 18 (7) 4 (1)	

^a^AI: artificial intelligence.

^b^Absolute frequency.

^c^Relative frequency.

A deep dive into AI capabilities revealed that 96% (n=257) of services can recognize patterns within audio, video, or other health-related data. At 33% (n=89), the second most frequent AI capability is to generate something new based on available input data. Examples are structured consultation protocols based on sound recordings or descriptions of injuries based on image files. Approximately 28% (n=75) of observed services include the AI capability to perform decision-making, for instance, to decide between multiple treatment alternatives. Another 13% (n=34) of observed services have the AI capability of reasoning, which offers the medical professional the significant benefit of delegating the task of explaining the link between bad habits and potential diseases. The rarest observed AI capabilities were acting (n=30, 11%), for example, calling emergency staff for help, and predicting (n=28, 10%), for example, being able to tell in advance whether people with specific characteristics will develop a particular disease. The final statistic of note is that most of our sample services are used for diagnostics (n=163, 61%). Second in demand (n=83, 31%) is the type of service used in treatment and care. At 87% (n=231), the vast majority of our sample services are used to treat physical illness or injury. In comparison, 9% (n=25) are used for medical triage. Only a minority of our sample services use AI to improve mental (n=18, 7%) or social (n=4, 1%) health.

### Archetypes and Interpretation

To ensure that our taxonomy can identify separable archetypes despite its extensive range, we performed the cluster analysis analogous to Gimpel et al [27], which is to say that we performed it for each of the identified dimensions separately. This cluster analysis revealed 13 archetypes of AI-based health care services that differ in their dominant design characteristics. We identified 2 archetypes in the agent perspective, 5 archetypes in the data perspective, 2 archetypes in the AI perspective, and 4 archetypes in the health impact perspective. Table 6 presents the respective number of services that can be assigned to each archetype as well as the absolute and relative frequencies of their characteristics.

First, the agent perspective includes the 2 archetypes, “AI-initiated” and “recipient-initiated.” Service offerings of the AI-initiated archetype actively request information from the user. They are designed for use by both the patient (27/57, 47%) and the health care professional (35/57, 61%). In contrast, the recipient-initiated archetype targets the medical professional in the majority of cases (152/211, 72%).

Second, the data perspective includes 5 archetypes. These mainly differ from one another in the origin and the type of the used data. What the archetypes have in common, however, is that their data are primarily related to the patient. The “structured object” archetype is characterized by the use of structured data (100%) that are provided by an object (100%). Almost one-quarter of services also use the patient (8/44, 18%) or medical professionals (3/44, 7%) as a source of information. In contrast, services in the “unstructured object” archetype work with unstructured data (100%) that is primarily available in audio or image formats. Occasionally, this data include information about the patient’s environment (13/145, 9%) but rarely about the responsible medical personnel (1/145, 1%). The “structured nonobject” archetype contains structured data concerning the patient (100%). These data are primarily provided by the patient (32/34, 94%) and less so by the treating physician (6/34, 18%). Services of the “unstructured nonobject” archetype work exclusively with unstructured data (100%). However, compared to the “structured nonobject” archetype, they are almost twice as likely (9/28, 32%) to be generated by the responsible health care professional. As for the “structured-unstructured” archetype, this one is characterized by the simultaneous processing of structured and unstructured data (100%) that are obtained from objects (12/17, 71%), health care professionals (8/17, 47%), and patients (7/17, 41%).

Third, the AI perspective comprises the 2 archetypes, “device-dependent” and “device-independent.” The former includes the majority of services in the sample (222/268, 82.8%) and operates with generic hardware. Services belonging to this archetype are primarily offered as stand-alone solutions (206/222, 93%). The underlying AI can recognize patterns and other correlations (215/222, 97%), decide between separate alternatives in approximately one-third of the cases (64/222, 29%), or generate output (79/222, 36%). To a lesser extent, it is also able to provide reasons for correlations (27/222, 12%), make predictions (23/222, 10%), and execute actions on its own (24/222, 11%). Services of the “device-dependent” archetype require the use of application-specific hardware. If supported by such hardware, this archetype can recognize contexts in a similar number of cases (44/46, 96%) as the previous archetype, but it makes decisions (10/46, 22%) or generates objects (10/46, 36%) in far fewer cases.

Fourth, the health impact perspective comprises 4 archetypes: “physical,” “diagnostic-physical,” “treatment,” and “triage.” Health care applications of the “physical” archetype provide material benefits to the patient (30/40, 75%). These are often achieved by means of preventive measures (28/40, 70%) or measures that help the patient to be more independent and self-determined in everyday life (11/40, 28%). Services of the “diagnostic-physical” archetype also focus on physical complaints (100%). In contrast to the previous archetype, however, they almost exclusively aim at disease diagnosis (131/132, 99%). Typically, services classified as belonging to the “treatment” archetype are used after the patient has been diagnosed (75/76, 99%). Another typical feature of these services is that they have a physical benefit in as much as 87% (66/76) of cases and a psychological benefit in as little as 13% (10/76). As for services of the “triage” archetype, these address patient referral to the appropriate health care specialist (100%). Given the comparatively small number of services in our sample (n=20), the underlying data may not be representative of real-world services. However, in three-quarters (15/20, 75%) of the cases, the occurrence of this archetype is closely linked to the diagnostic process. Other use cases are found mainly in the prevention (4/20, 20%) and treatment (9/20, 45%) of previously diagnosed diseases.

**Table 6:** Archetypes of 268 AI^a^-based health care services.

Archetype and dimension				Characteristics		Frequency (N=268), n^b^ (%^c^)
**Agents**						
	**AI-initiated (n=57)**					
		Service recipient	Patient Health care professional		27 (47.4) 35 (61.4)	
		Mode of interaction	AI-driven Recipient-driven		57 (100) 0 (0)	
	**Recipient-initiated (n=211)**					
		Service recipient	Patient Health care professional		69 (32.7) 152 (72)	
		Mode of interaction	AI-driven Recipient-driven		0 (0) 211 (100)	
**Data**						
	**Structured object (n=44)**					
		Data generator	Patient Health care professional Object		8 (18.2) 3 (6.8) 44 (100)	
		Data target	Patient Health care professional Environment		42 (95.5) 2 (4.5) 1 (2.3)	
		Data type	Structured Unstructured		44 (100) 0 (0)	
	**Unstructured object (n=145)**					
		Data generator	Patient Health care professional Object		4 (2.8) 1 (0.7) 145 (100)	
		Data target	Patient Health care professional Environment		137 (94.5) 3 (2.1) 13 (9)	
		Data type	Structured Unstructured		0 (0) 145 (100)	
	**Structured nonobject (n=34)**					
		Data generator	Patient Health care professional Object		32 (94.1) 6 (17.6) 0 (0)	
		Data target	Patient Health care professional Environment		34 (100) 0 (0) 5 (14.7)	
		Data type	Structured Unstructured		34 (100) 0 (0)	
	**Unstructured nonobject (n=28)**					
		Data generator	Patient Health care professional Object		22 (78.6) 9 (32.1) 0 (0)	
		Data target	Patient Health care professional Environment		26 (92.9) 2 (7.1) 3 (10.7)	
		Data type	Structured Unstructured		0 (0) 28 (100)	
	**Structured-unstructured** **(n=17)**					
		Data generator	Patient Health care professional Object		7 (41.2) 8 (47.1) 12 (70.6)	
		Data target	Patient Health care professional Environment		17 (100) 0 (0) 0 (0)	
		Data type	Structured Unstructured		17 (100) 17 (100)	
**AI**						
	**Device-independent (n=222)**					
		Hardware agnostic	Yes No		222 (100) 0 (0)	
		AI portfolio integration	Wrapped around product Wrapped around service Stand-alone solution		1 (0.5) 15 (6.8) 206 (92.8)	
		AI capability	Recognizing Reasoning Predicting Decision-making Generating Acting		215 (96.8) 27 (12.2) 23 (10.4) 64 (28.8) 79 (35.6) 24 (10.8)	
	**Device-dependent** **(n=46)**					
		Hardware agnostic	Yes No		0 (0) 46 (100)	
		AI portfolio integration	Wrapped around product Wrapped around service Stand-alone solution		9 (19.6) 0 (0) 37 (80.4)	
		AI capability	Recognizing Reasoning Predicting Decision-making Generating Acting		44 (95.7) 6 (13) 5 (10.9) 10 (21.7) 10 (21.7) 7 (15.2)	
**Health Impact**						
	**Physical (n=40)**					
		Application area	Prevention Diagnostics Treatment and care Patient enablement		28 (70) 1 (2.5) 1 (2.5) 11 (27.5)	
		Health benefit	Triage Physical Mental Social		1 (2.5) 30 (75) 6 (15) 3 (7.5)	
	**Diagnostic-physical (n=132)**					
		Application area	Prevention Diagnostics Treatment and care Patient enablement		18 (13.6) 131 (99.2) 0 (0) 0 (0)	
		Health benefit	Triage Physical Mental Social		5 (3.8) 132 (100) 2 (1.5) 0 (0)	
	**Treatment (n=76)**					
		Application area	Prevention Diagnostics Treatment and care Patient enablement		5 (6.6) 17 (22.4) 75 (98.7) 2 (2.6)	
		Health benefit	Triage Physical Mental Social		0 (0) 66 (86.8) 10 (13.2) 1 (1.3)	
	**Triage (n=20)**					
		Application area	Prevention Diagnostics Treatment and care Patient enablement		4 (20) 15 (75) 9 (45) 3 (15)	
		Health benefit	Triage Physical Mental Social		20 (100) 4 (20) 0 (0) 0 (0)	

^a^AI: artificial intelligence.

^b^Absolute frequency.

^c^Relative frequency.

In summary, the agent archetypes differ mainly in how and by whom information is requested. It is worth noting that approximately 8 (80%) out of 10 services require manual process initiation, which means they need human interaction. This reliance on the interaction between humans and AI is also predicated on the fact that both archetypes are directed at trained health care professionals in more than half the cases. When it comes to the “data” perspective, we found the distinguishing characteristics to be the type of data and the data generator. Potentially, this can be explained by the fact that all 5 archetypes focus predominantly on patients as data targets. This is logical since information about a patient’s complaints, symptoms, or medical history concerns that patient directly. It is questionable, however, whether AI-based applications in the health care sector are still making insufficient use of some of the available data, for example, environmental information about a patient’s working or living conditions. As for the archetypes of the “AI” perspective, they distinguish between health care service offerings according to their dependency on specific hardware. This is consistent with the aforementioned high proportion of applications for image file interpretation, as these typically do not involve hardware components. Another reason for the high proportion of device-independent applications could be the complexity and cost of product development. This brings us to the final point—the archetypes in the “health impact” perspective. Their analysis focuses on patient benefit and shows that, to date, AI-based applications are primarily used for the diagnosis of physical illnesses. Given the increasing life expectancy of most industrialized nations, it is conceivable that the importance of AI in treatment and care, especially in geriatric care, might grow considerably in the near future. As for the other seismic shift in the technologically advanced West—our rapidly increasing awareness of mental health as a medical, social, and indeed political issue, it remains to be seen whether the number of services for the diagnosis and treatment of mental illnesses will also increase in the future.

## Discussion

### Principal Findings

To account for the phenomenon of AI-based health care services, we developed a new taxonomy based on the latest science [24], a structured literature review, and an extensive sample of real-world instantiations. As our rigorous demonstration and evaluation confirmed, this due diligence in the development of our taxonomy ensured its broad applicability and central positioning in the intended research field. As a result, this study contributes to the knowledge of how to incorporate AI into health care services successfully. Furthermore, it provides a clear and comprehensive structure to a fast-developing research field. Although there may be questions about the usefulness of taxonomy in such a dynamic field, this study indicates that a phenomenon does not have to be static for it to benefit from a taxonomy. As we have seen in similarly fast-developing research areas, such as the Internet of Things [26], FinTechs [27], analytics-based services [44], or Blockchain [74], there have been considerable benefits as a result of taxonomy development.

Meanwhile, our research results have theoretical as well as managerial implications. From a theoretical point of view, our research is the first to investigate the structure of AI-based health care services based on perspectives, dimensions, respective characteristics, and overall archetypes. The taxonomy supports academics and practitioners alike in that it enables them to recognize and re-evaluate the multiple forms of AI-based health care services, either to use those services to their full practical potential or to use the taxonomy for further theorizing. The archetypes we have identified in each perspective reveal various service offerings to have recurring patterns. These patterns advance the current understanding of how digital technologies transform the health care sector with the use of AI. Most importantly, this study incentivizes more extensive research on application areas in which AI has not yet been used to its full potential. Doing so encourages scholars to explore the multiple opportunities to apply AI in the ever more significant sector of digital health care.

From a practical point of view, our taxonomy and archetypes provide multiple use cases for practitioners. First, it is worth noting that practitioners who have already integrated AI into their health care service may find our taxonomy helpful in classifying this service and comparing it to any other offerings by their competitors. Second, practitioners who wish to introduce new AI-based health care services can use our taxonomy to gain an overview of possible applications, be it to find suitable options that are already out there or to find inspiration for potential new applications in their working fields. By analyzing numerous real-world examples, we were able to shed light on the intricacies of AI-based health care services and the general distribution of their design characteristics. By using our archetypes along with our insights into their frequencies, practitioners can identify which potential AI adoption fields are already widely used and might, therefore, be easier and more accessible areas in which to implement their services. While this focus provides limited potential for future innovations, our taxonomy facilitates the assessment of alternative possibilities in less common adoption fields, where it can be used for the dual benefits of orientation and structure. Third, the insights derived from our taxonomy on the characteristics of AI-based health care services carry substantial implications for health policy and delivery. By categorizing AI services based on their unique features, policy makers can make informed decisions on resource allocation, regulation, and integration into existing health care systems. This taxonomy facilitates the identification of targeted interventions for improving patient care, such as personalized treatment plans and enhanced diagnostic accuracy. Fourth, understanding the characteristics of AI-based services aids in the development of policies that address ethical considerations, privacy concerns, and the need for transparency in health care AI implementation. Fifth, in the context of population health, the taxonomy enables the strategic deployment of AI technologies to address specific health challenges, reduce disparities, and optimize overall health outcomes. This discussion underscores the practical relevance of our taxonomy, extending beyond theoretical considerations to directly influence health care policy making and health care delivery.

Of course, this study is also subject to certain limitations that require careful consideration to ensure the correct interpretation of our results. The first of those limitations to address is that our classification, as to whether or not a service is AI-driven, is based on the external appearance given by the service provider’s website or information supplied by other media. It cannot, therefore, be guaranteed that all of the services considered in this study meet the academic criteria to be classified as AI-driven. There is a hidden benefit in this putative limitation, however. Our research approach does not focus on how AI is seen in research but rather on how AI is perceived and applied in practice. The second limitation to discuss is that AI is a rapidly evolving field, and as new developments overtake the research presented in these pages, our taxonomy may need to be adapted and extended accordingly. For example, the sample of service offerings that we analyzed has a noticeably high frequency of diagnostics-related services due to our definition of the term service and AI algorithms that were primarily used for making decisions and classifying symptoms. However, current developments in the topics of generative AI or robotics might shift this trend toward treatment and care or patient enablement. With that in mind, we were especially thorough in our structured analysis of current AI adoption and emphasized the extensibility of our taxonomy to provide the necessary guidance for future adaptations and extensions in research and practice. The third limitation worth considering is that we based our research on empirical insights from top lists. While our data collection was rigorous, our selection of the publicly available AI-based health care services only represents a subset of the current services. As for the fourth limitation of this study, while we are confident that our taxonomy offers a good representation of the current AI landscape in health care, it will undoubtedly need to be changed and adapted due to rapid developments at a technical level [75]. This could particularly affect the dimensions of AI capability and the combination with the application area. However, the most significant change will probably be seen in the archetypes. A comparison between the current and future status would undoubtedly be interesting for future research.

### Conclusions and Outlook

Being a relatively new paradigm in health care, AI has been heralded as a panacea, yet only the future will tell if it can deliver on those lofty expectations. In the meantime, we have developed a taxonomy that serves as a structuring tool for researchers in the field of AI and as an innovation map for practitioners. It provides a simple yet potent method of classifying current and future AI-based health care services. Such classification allows the user to better situate and evaluate these services by attributing their distinct characteristics to 10 potential dimensions, such as service recipient, mode of interaction, data generator, data target, data type, hardware agnostic, AI portfolio integration, AI capability, application area, and health benefit. Having tested our taxonomy with representatives of its intended future users in order to classify 268 AI-based health care services and identify all relevant archetypes, we were able to ascertain its applicability and assess the potential for AI integration in current health care services.

By way of conclusion, then, it remains only to be said that this study was conducted in the intellectual tradition of Nickerson et al [24] and Kundisch et al [25]. As such, it contributes to the expert literature by providing a common understanding of AI-based health care services and offering a taxonomy that promises to assist in the development of AI-based health care services and the assessment of their potential in this competitive field. It bears repeating, however, that the implementation of AI in health care is a young and rapidly developing research field, so although we are convinced that this taxonomy can serve as a useful analytical tool in the present, its future usefulness will depend on its adaptation to relevant advances in the field. We hope that our work here will inspire the future research required to identify those advances and make the necessary adaptations to our taxonomy.

## Appendix

Multimedia Appendix 1 Taxonomy evaluation and clustering.

## Abbreviations

AI: artificial intelligence
C2E: conceptual-to-empirical
E2C: empirical-to-conceptual
