# Supplementary material for: A Taxonomy and Archetypes of AI-Based Health Care Services: Qualitative Study
Source: J Med Internet Res. 2024 Nov 27;26:e53986. doi: 10.2196/53986 (PMC11635336; doi:10.2196/53986)
Supplement: Multimedia Appendix 1 [file jmir_v26i1e53986_app1.docx]

## Taxonomy Evaluation

First, the percentage agreements per dimension are determined, i.e., the number of correct answers is measured against the total number of answers (Cohen 1960). The results are then examined in more detail by using the so-called kappa coefficient as a more exact measure of interrater reliability (Fleiss 1971). The term is best explained by Nahm et al. (2002) as the degree of “agreement after chance agreement is excluded”. Fleiss’ (1971) kappa builds on the kappa originally developed by Cohen (1960), in which two fixed independent raters assess a number of units with nominally scaled categories. As an extension to the original concept, Fleiss’ kappa can be used to determine the degree of agreement between the assessments of more than two raters, provided that the number of raters per assessed unit remains constant. The calculation of Fleiss’ kappa requires nominal or ordinal categories. The unweighted Fleiss’ kappa is determined, i.e., each non-match is weighted equally. The calculation of Fleiss’ kappa (1) results from the calculation of the relative agreement of the raters (2) and the calculation of the probability of random agreement (3).

(1) $\kappa= \frac{p_{0}- p_{e}}{1- p_{e}}$

(2) $p_{0}= \frac{1}{N} \sum_{i=1}^{N} (\frac{1}{n\left( n-1 \right)} \sum_{j=1}^{k} (n_{ij}^{2}-n_{ij}))$

(3) $p_{e}= \sum_{j=1}^{k} p_{j}^{2}$

$n$ describes the number of raters ($n$ = 13, see chapter 4.1), N the number of considered service offerings per rater (N = 4), and k the number of possible evaluation categories ($k$ = 2 for Service Recipient, Mode of Interaction, Data Type, Hardware Agnostic; $k$ = 3 for Data Generator, Data Target, AI Portfolio Integration; $k$ = 4 for Application Area, Health Benefit; $k$ = 6 for AI Capability).

See Table 1 for a breakdown of the inter-coder reliability as percentage agreement and as values of Fleiss’ kappa. Across all dimensions and participants, the percentage agreement is 88%, which means that in almost nine out of ten cases the objects are assigned to the same category. The lowest value is achieved in the dimension *Hardware Agnostic* (77%), the highest in the dimension *Application Area* (100%). In six of the ten dimensions, there is a match of at least 85%, in four of them, this match rate is 90% or more.

Table S1. Inter-coder reliability for all ten dimensions as percentage agreement and as Fleiss’ (1973) kappa.

| **Dimension** | **Percent agreement** | **Fleiss` kappa** |
| --- | --- | --- |
| *Service Recipient* | 0.9615 | 0.8452 |
| *Mode of Interaction* | 0.8846 | 0.5615 |
| *Data Generator* | 0.8974 | 0.6872 |
| *Data Target* | 0.9615 | 0.8452 |
| *Data Type* | 0.8077 | 0.3294 |
| *Hardware Agnostic* | 0.7692 | 0.2308 |
| *AI Portfolio Integration* | 0.8077 | 0.5117 |
| *AI Capability* | 0.8718 | 0.7234 |
| *Application Area* | 1.0000 | 1.0000 |
| *Health Benefit* | 0.8077 | 0.4506 |
| **Average** | **0.8769** | **0.6185** |

The calculation of Fleiss’ kappa results in a value of k = 0.6185, equally weighted among all ten dimensions. For the individual dimensions, resulting values range between k = 0.3294 for the dimension *Hardware Agnosti*c and k = 1 for the dimension *Application Area*.

## Clustering

To determine an appropriate number of clusters, we calculated a number of measures discussed in the current literature for the present data set. As shown in Table 2, values from 1 to 20 result for the recommended number of optimal clusters. In the absence of a clear result and a recognised standard in the research field of performance and validation measures for cluster analyses (Wu 2012), the dimensions of the individual perspectives are considered in groups and several cluster solutions are created for each perspective. In addition to the number of services in each cluster and the manageability of the resulting overall solution, the criterion of interpretability is considered when determining the number of clusters (Milligan and Cooper 1985; Sneath and Sokal 1973).

Table S2. Suggested number of clusters according to 17 measures.

| **Clustering validation measures** | |
| --- | --- |
| **Measure suggested by** | **Suggested number of clusters** |
| *Dunn (1974)* | 15 |
| *Krzanowski and Lai (1988)* | 9 |
| *Calinski and Harabasz (1974)* | 2 |
| *Hartigan (1975)* | 9 |
| *Hubert and Levin (1976)* | 20 |
| *Davies and Bouldin (1979)* | 19 |
| *Rousseeuw (1987)* | 5 |
| *Ratkowsky and Lance (1978)* | 4 |
| *Ball and Hall (1965)* | 3 |
| *Milligan (1980), Milligan (1981)* | 5 |
| *Tibshirani et al. (2001)* | 5 |
| *Frey and van Groenewoud (1972)* | 1 |
| *McClain and Rao (1975)* | 2 |
| *Baker and Hubert (1975)* | 20 |
| *Rohlf (1974), Milligan (1981)* | 20 |
| *Halkidi et al. (2000)* | 8 |
| *Halkidi and Vazirgiannis* | 20 |
